# Ectopic pancreas: A rare cause of occult gastrointestinal bleeding

**DOI:** 10.1016/j.amsu.2020.09.005

**Published:** 2020-09-09

**Authors:** Houssem Ammar, Mohamed Amine Said, Abdelkader Mizouni, Waad Farhat, Fathia Harrabi, Linda Ghabry, Rahul Gupta, Mohamed Ben Mabrouk, Ali Ben Ali

**Affiliations:** a University of Sousse, Department of Gastrointestinal Surgery, Sahloul Hospital, Sousse, Tunisia; b Department of Gastrointestinal Surgery, Synergy Institute of Medical Sciences, Dehradun, India

**Keywords:** Ectopic pancreas, Heterotopic pancreas, Jejunum, Surgery

## Abstract

Ectopic pancreas (EP) is a rare entity characterized by the development of pancreatic tissue in areas other than the pancreas. We present the case of a 16-year-old female with a heterotopic pancreas in the jejunum revealed by occult gastrointestinal bleeding. Contrast-enhanced computed tomography (CT) of the abdomen revealed a 2 × 3 cm enhancing nodular jejunal mass suspicious of a neuroendocrine or gastrointestinal stromal tumor. Octreoscan was planned but the patient presented in the emergency department with fever and sudden onset severe abdominal pain. The patient underwent emergency laparotomy. On abdominal exploration, appendicular perforation was present for which appendectomy and peritoneal lavage were performed. The small jejunal lesion seen on CT was identified during surgery and segmental jejunal resection with end-to-end anastomosis was performed. The histopathological examination of the jejunal mass revealed the presence of pancreatic acini and ductal structures without islets of Langerhans in the submucosa of the small intestine covered by normal mucosa. At the last follow-up of eight months after surgery, the patient is symptom-free and the abdominal CT is normal. Preoperative diagnosis of EP requires high clinical suspicion and should be included in the differential diagnosis while treating patients with gastrointestinal bleeding or gastrointestinal mass on CT.

## Highlights

• Ectopic pancreas (EP) is a rare entity characterized by the development of pancreatic tissue in areas other than the pancreas.
• It is often asymptomatic and mostly detected incidentally on surgery for other disease or on autopsy.
• The most common sites of EP are stomach and small intestine but it can be found in the esophagus, ileum, and biliary tree.
• EP should be included in the differential diagnosis while treating patients with gastrointestinal bleeding or gastrointestinal mass on CT.

## 1 Introduction

Ectopic pancreas (EP) is a rare entity characterized by the development of pancreatic tissue in areas other than the pancreas and without any ductal or vascular continuity [1].

It is often asymptomatic and mostly detected incidentally on surgery for other diseases or on autopsy. The reported incidence of EP at autopsy is 0.6–14% and it is estimated to be incidentally detected during 0.2% of upper abdominal surgeries [2]. The most common sites of EP are the stomach, duodenum and small intestine but it can be found in the esophagus, ileum, and biliary tree [3]. We present the case of a 16-year-old female with a heterotopic pancreas in the jejunum revealed by occult gastrointestinal bleeding. This case has been reported in line with the SCARE criteria [4].

### 1.1 Case description

**Fig. 1:**
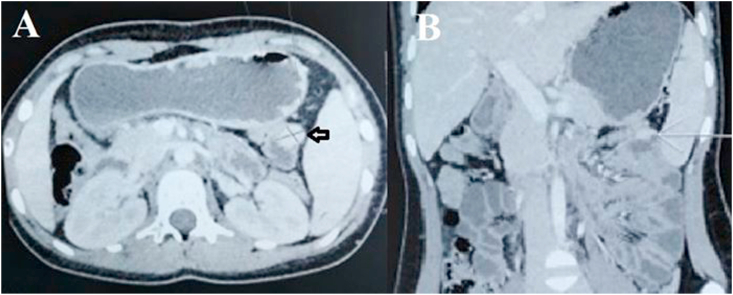
Contrast enhanced computed tomography showing the heterogeneously enhancing lesion in the jejunal wall (arrow) on axial (A) and coronal (B) sections.

**Fig. 2:**
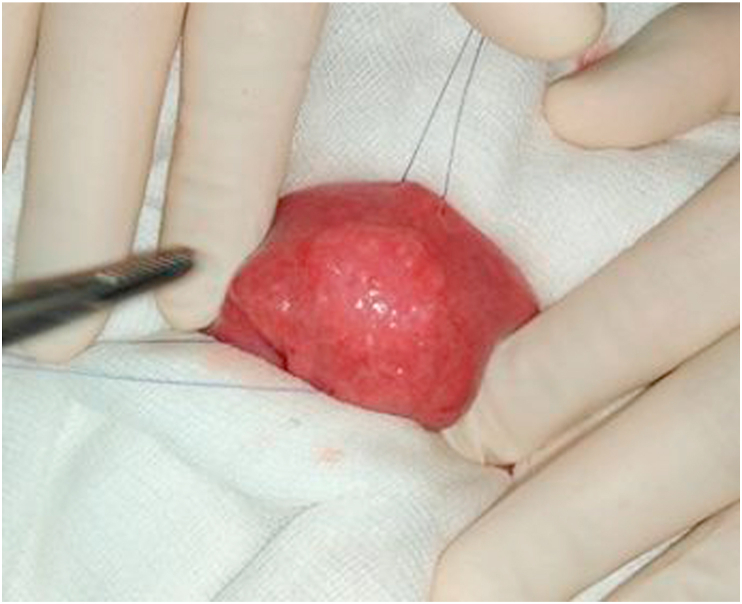
Intraoperative photograph showing the jejunal lesion.

**Fig. 3:**
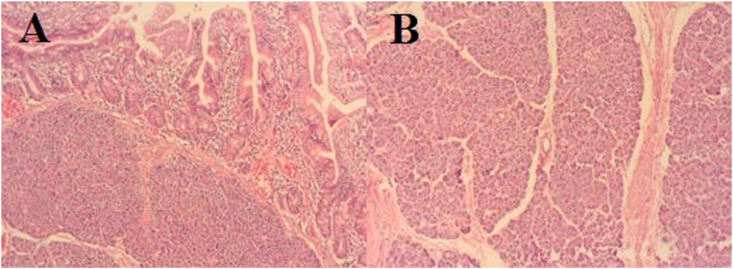
Microscopic examination of the jejunal lesion revealed the presence of heterotopic pancreas in the jejunal wall (A) evident by the well-formed pancreatic acini with minimally developed ducts (B). (H & E, 20x).

A 16-year-old girl presented with anemia and melena for two months. The hemoglobin level was 9 gm/dL. Stool for occult blood was positive. Upper gastrointestinal endoscopy and colonoscopy were normal. Contrast-enhanced computed tomography (CT) of the abdomen revealed a 2 × 3 cm enhancing nodular jejunal mass suspicious of neuroendocrine or gastrointestinal stromal tumor (Fig. 1). Octreoscan was planned but the patient presented in the emergency department with fever and sudden onset severe abdominal pain. On clinical examination, there were signs of peritonitis. Laboratory investigations revealed leukocytosis and high C-reactive protein levels. CT abdomen revealed acute appendicitis with suspected perforation. The patient underwent emergency laparotomy. On abdominal exploration, appendicular perforation was present for which appendectomy and peritoneal lavage was performed. The small jejunal lesion seen on CT was identified during surgery and segmental jejunal resection with end-to-end anastomosis was performed (Fig. 2). The postoperative course was uneventful and the patient was discharged after 5 days. The histopathological examination of the jejunal mass revealed the presence of pancreatic acini and ductal structures without islets of Langerhans in the submucosa of the small intestine covered by normal mucosa. There was no evidence of malignancy. The final diagnosis of heterotopic pancreas Type 2 as per Heinrich classification^1^ was made (Fig. 3). At the last follow-up of eight months after surgery, the patient is symptom-free and the abdominal CT is normal.

## 2 Discussion

Ectopic (EP) or heterotopic pancreas is a rare disorder characterized by the presence of pancreatic parenchymal tissue outside its usual anatomical location without any structural or vascular continuity with the main pancreas [2]. It The true incidence of heterotopic pancreas is difficult to determine, as most affected patients are asymptomatic. Most of EP lesions do not cause symptoms and are incidentally detected during abdominal surgeries for other indications [3]. The frequent complications associated with EP include gastrointestinal bleeding, development of intussusception, bowel obstruction and bowel perforation [1,5]. Rarely, malignancy such as adenocarcinoma, intraductal papillary mucinous neoplasm and solid pseudopapillary tumor can develop in the EP [6,7].

Preoperative diagnosis of EP is difficult. They appear as mural wall thickening, exophytic or endoluminal enhancing lesions on CT as seen in the present case [8]. On endoscopy, they appear as a circular to oval submucosal lesion with central umbilication [9]. However, definitive diagnosis can be made on histological examination only. Surgical resection is the treatment of choice for both symptomatic and incidental cases of EP in order to prevent future complications [6]. The surgery can be performed by open or laparoscopic techniques depending on the location of the lesions and the available surgical expertise.

Histologically, the lesions will show the presence of pancreatic acini, ducts and/or islet cells. They can be classified in to four types depending on the pancreatic elements present in the lesion as described by Heinrich et al. [3,7]. In the current case, the pancreatic acini and ducts were seen and classified as Type 2 EP.

## 3 Conclusion

Preoperative diagnosis of EP requires high clinical suspicion and should be included in the differential diagnosis while treating patients with gastrointestinal bleeding or gastrointestinal mass on CT.

## Declaration of competing interest

The authors declare that they have no conflict of interest.

## Registration of research studies

1.Name of the registry:

2.Unique Identifying number or registration ID:

3.Hyperlink to the registration (must be publicly accessible):

As this was a case report and not a clinical trial, this study does not require registration.
